# Radioisotope-Guided Sentinel Lymph Node Biopsy in Penile Cancer: A Long-Term Follow-Up Study

**DOI:** 10.3389/fonc.2022.850905

**Published:** 2022-04-14

**Authors:** Lena Nemitz, Anna Vincke, Bianca Michalik, Svenja Engels, Luca-Marie Meyer, Rolf-Peter Henke, Friedhelm Wawroschek, Alexander Winter

**Affiliations:** ^1^University Hospital for Urology, Klinikum Oldenburg, Department of Human Medicine, School of Medicine and Health Sciences, Carl von Ossietzky University Oldenburg, Oldenburg, Germany; ^2^Institute of Pathology Oldenburg, Oldenburg, Germany

**Keywords:** penile cancer, sentinel lymph node, dynamic sentinel node biopsy, inguinal lymphadenectomy, lymph node metastases

## Abstract

Lymph node (LN) management is critical for survival in patients with penile cancer. However, radical inguinal lymphadenectomy carries a high risk of postoperative complications such as lymphedema, lymphocele, wound infection, and skin necrosis. The European Association of Urology guidelines therefore recommend invasive LN staging by modified inguinal lymphadenectomy or dynamic sentinel node biopsy (DSNB) in clinically node-negative patients (cN0) with intermediate- and high-risk tumors (≥ T1G2). However, the timing of DSNB (simultaneous vs. subsequent to partial or total penile resection) is controversial and the low incidence of penile cancer means that data on the long-term outcomes of DSNB are limited. The present study aimed to analyze the reliability and morbidity of DSNB in patients with penile cancer during long-term follow-up. This retrospective study included 41 patients (76 groins) who underwent radioisotope-guided DSNB simultaneously or secondarily after penile surgery from June 2004 to November 2018. In total, 193 sentinel LNs (SLNs) and 39 non-SLNs were removed. The median number of dissected LNs was 2.5 (interquartile range 2–4). Histopathological analysis showed that five of the 76 groins (6.6%) contained metastases. None of the non-SLNs were tumor-positive. In accordance with the guidelines, all inguinal regions with positive SLNs underwent secondary radical inguinal lymphadenectomy, which revealed three additional metastases in one groin. Regional LN recurrence was detected in three patients (four groins) during a median follow-up of 70 months, including two patients in whom DSNB had been performed secondarily after repetitive penile tumor resections. DSNB-related complications occurred in 15.8% of groins. Most complications were mild (Clavien–Dindo grade I; 50%) or moderate (II; 25%), and invasive intervention was only required in 3.9% of groins (IIIa: *n* = 1; IIIb: *n* = 2). In summary, this study suggests that the current radioisotope-guided DSNB procedure may reduce the complication rate of inguinal lymphadenectomy in patients with cN0 penile cancer. However, DSNB and penile surgery should be performed simultaneously to minimize the false-negative rate. Recent advances, such as new tracers and imaging techniques, may help to reduce the false-negative rate of DSNB further.

## Introduction

Penile cancer is a rare disease with an overall incidence of less than one case in 100,000 persons worldwide (1). Lack of knowledge about the disease and the feeling of embarrassment often lead to a delay in diagnosis (2). The most important prognostic factor in patients with penile carcinoma is the presence of lymph node (LN) metastases (3, 4). Metastatic spread of penile cancer typically occurs in a stepwise fashion with the inguinal LNs affected first, followed by spread to the pelvic and distant LNs (5). An analysis of 944 patients with penile squamous cell carcinoma revealed that patients without nodal involvement had a 5-year cancer-specific survival rate of 90%, but this rate was considerably reduced to 56% in patients with LN metastases (6). Further studies showed that early “prophylactic” inguinal lymphadenectomy improved survival compared with delayed lymphadenectomy when metastases became clinically evident (7, 8). The management of regional LNs is thus essential in the treatment of penile cancer.

According to the European Association of Urology (EAU) guidelines on penile cancer, the management of LNs depends on the clinical LN status (9). Patients with palpable inguinal LNs are at high risk of lymphatic spread, and radical inguinal lymphadenectomy is therefore indicated in these patients. However, the optimal management of regional LNs in patients with clinically normal LNs (cN0) is more controversial. Approximately 20%–25% of these patients harbor occult LN metastases (10–12). Unfortunately, current imaging modalities, such as computed tomography, positron emission tomography/computed tomography, and magnetic resonance imaging cannot reliably detect micrometastases (13). Clinical surveillance of cN0 patients carries the risk of not detecting metastases until a later stage, with a negative effect on patient prognosis (7, 8). In contrast, radical inguinal lymphadenectomy is associated with a high rate of complications, such as skin-edge necrosis, wound infection, seroma, and lymphedema, and may result in overtreatment in 75%–80% of these patients (14, 15). The EAU guidelines thus recommend invasive LN staging by either modified inguinal lymphadenectomy or dynamic sentinel node biopsy (DSNB) for cN0 patients with intermediate- (pT1G2) or high-risk (≥ T1G3) tumors.

Modified inguinal lymphadenectomy aims to reduce the morbidity associated with radical inguinal lymphadenectomy by limiting the dissection area and preserving the saphenous vein (16–23). However, the false-negative rate of modified inguinal lymphadenectomy is unknown (9).

The concept of sentinel node biopsy was first described by Cabañas more than 40 years ago (24). This method relies on the principle that the first LNs on the direct drainage pathway of a tumor, referred to as the sentinel LNs (SLNs), will be the first sites of metastasis. Based on this assumption, a negative SLN biopsy indicates the absence of lymphatic spread and radical inguinal lymphadenectomy can thus be avoided. Using lymphangiography, Cabañas identified a LN at the anterior or medial aspect of the superficial epigastric vein as the SLN for the penis. However, consideration of this static model resulted in a large number of false-negative results (25–27). In 2000, the concept of DSNB was introduced in cases of penile cancer (28, 29). DSNB enabled the individual patient’s SLNs to be identified by peritumoral injection of a radioactive tracer, preoperative lymphoscintigraphy, and intraoperative detection of radioactive LNs using a gamma probe. Continuous improvements of this method have reduced the complication and false-negative rates of DSNB to 5.7% and 4.8%, respectively (30). The reliability and morbidity of this technique have also been investigated in several other studies; however, most have included small patient numbers and reported highly variable complication and false-negative rates (31–37). The timing of DSNB is controversial. Two single-center studies suggested that DSNB was a reliable procedure for LN staging in cN0 patients after previous resection of the primary tumor (38, 39), while other authors observed regional recurrence after secondary, but not after primary DSNB, arguing against this hypothesis (40, 41).

We previously reported an initial experience of radioisotope-guided DSNB in patients with penile cancer in our center (42). A retrospective analysis of 32 patients with a median follow-up of 30.5 months revealed a complication rate of 11.1%, with no nodal recurrence. The present study aimed to update the outcomes of patients with penile cancer undergoing DSNB at our hospital, and to evaluate the reliability and morbidity of radioisotope-guided DSNB in a larger cohort with long-term follow-up.

## Materials and Methods

### Patients

**Figure 1:**
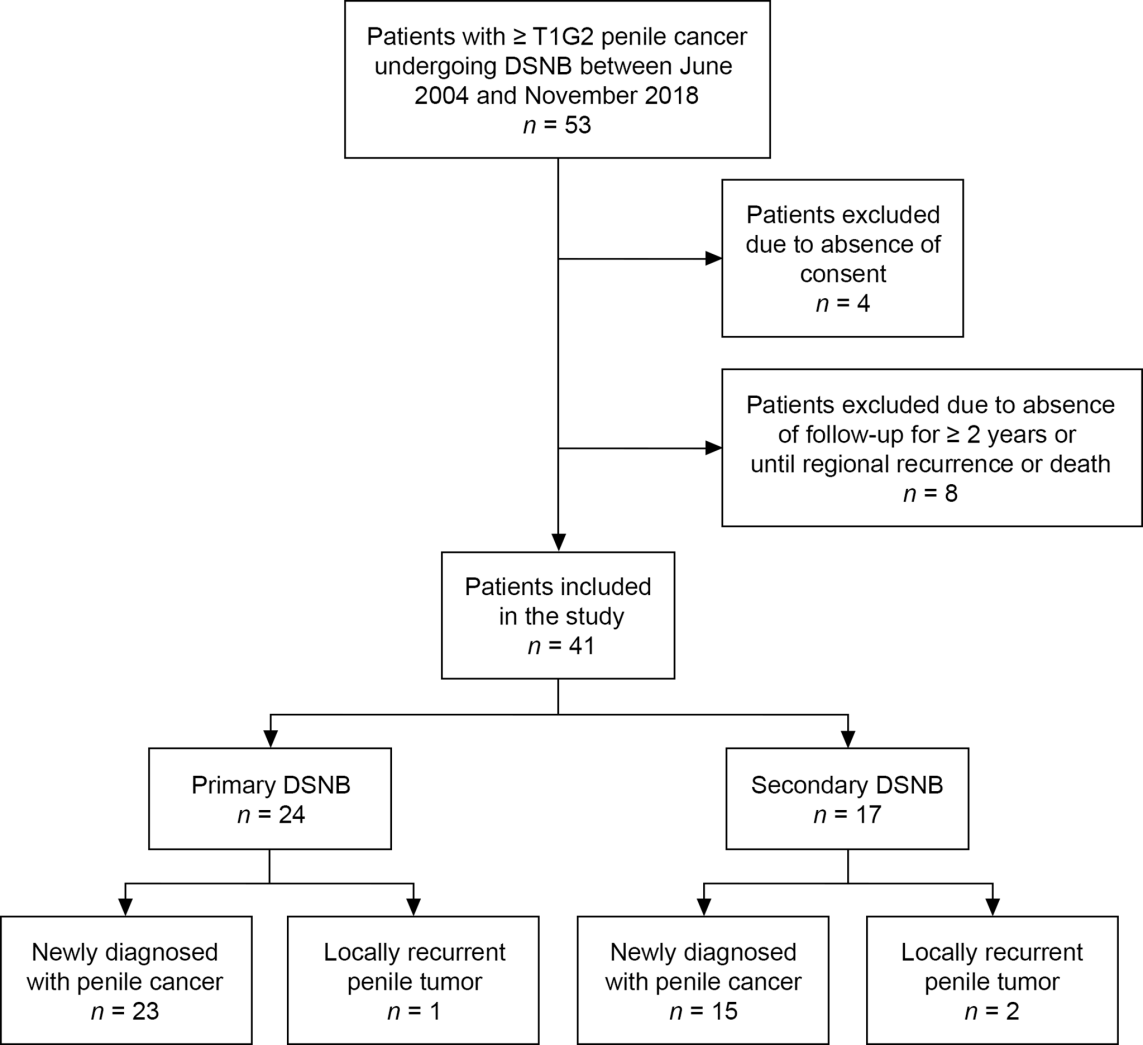
Flowchart of included and excluded patients.

Fifty-three patients with intermediate- or high-risk penile cancer (≥ T1G2) underwent radioisotope-guided DSNB at the University Hospital for Urology in Oldenburg, Germany, between July 2004 and November 2018. All patients were informed about DSNB verbally and in writing and provided signed consent. Four patients were excluded from this study because they did not want to participate. Another eight patients were excluded because they could not be followed up for at least 2 years or until regional recurrence or death. None of these patients developed tumor recurrence during follow-up. A total of 41 patients were left for analysis (**Figure 1**). Pre-existing cardiovascular diseases in the patient cohort were chronic rheumatic heart diseases, hypertension, coronary heart disease, myocardial infarction, peripheral atrial disease, cerebrovascular disease, stroke, and atrial fibrillation.

Of the 41 patients included in this study, 38 patients were newly diagnosed with penile cancer and three patients presented with recurrent tumors of the penis. The histological subtypes of penile tumors were categorized according to the respective current World Health Organization classification. Thirty-eight patients underwent surgical treatment of the primary tumor at our hospital, while three patients were initially treated in another hospital and referred to us for DSNB. DSNB was either performed during surgery for the primary tumor (*n* = 24) or as a secondary procedure (*n* = 17). Thirty-five patients underwent bilateral DSNB, and the remaining six patients received unilateral DSNB and unilateral modified or radical inguinal lymphadenectomy due to ipsilateral suspicious LNs (*n* = 2), histologically confirmed LN metastases (*n* = 2), or non-visualization of SLNs during DSNB (*n* = 2) in the same operation.

This study is registered in an international clinical trials register (Research Registry, researchregistry7492).

### Dynamic Sentinel Node Biopsy and Surgical Treatment

All patients underwent preoperative ultrasonography of both inguinal regions. ^99m^Technetium (^99m^Tc) nanocolloid (radioactivity ca. 30 Mbq) was injected peritumorally or in a two-step procedure into the resection area approximately 4 hours before surgery. Preoperative visualization of SLNs was achieved by lymphoscintigraphy. SLNs were detected intraoperatively using a gamma probe (C-Trak System, Care Wise, Morgan Hill, CA, USA; Crystal Probe SG04, Crystal Photonics GmbH, Berlin, Germany). Non-SLNs directly adjoining SLNs were also removed if *in situ* separation was not possible. Intraoperative palpation of the wound was also performed to identify and dissect clinically suspicious LNs.

In accordance with the EAU guidelines, patients with at least one positive LN were offered secondary ipsilateral radical inguinal lymphadenectomy and patients with at least two positive LNs were offered additional ipsilateral pelvic lymphadenectomy.

### Histopathological Examination

All dissected LNs were fixed in formalin, embedded in paraffin, and cut into 3-mm transverse sections. After deparaffinization and rehydration, 4- to 5-µm sections were stained with hematoxylin-eosin and examined by one of three pathologists with high experience in urological malignancies. If conventional histology was inconclusive, immunohistochemistry with a pancytokeratin antibody (AE1/AE3) was carried out using a DAKO Autostainer (Agilent Technologies, Santa Clara, CA, USA). In cases of false-negative DSNB results, SLNs were histopathologically reexamined by one pathologist.

### Definitions of Tumor Recurrences and Follow-Up

Tumor recurrences were classified into local, regional and distant recurrences. Local recurrence was defined as recurrent disease on the penis, regional recurrence was defined as recurrent disease in inguinal and/or pelvic LNs, and distant recurrence was defined as recurrent disease in distant LNs or other organs.

Follow-up was performed by resident urologists on an outpatient basis. Control visits were carried out at 3-, 6-, or 12-month intervals. Local or regional recurrence was detected by physical examination of the penis and groins, with ultrasound, computed tomography, or magnetic resonance imaging if indicated. The time of follow-up was defined as the time from DSNB to the latest follow-up, regional recurrence, or death of the patient. DSNB-related complications were assessed by analyzing hospital and outpatient clinical records and questionnaires completed by the patients and urologists. All complications were categorized according to the Clavien–Dindo classification (43).

### Analysis

DSNB was defined as a false-negative procedure if all SLNs were negative but non-SLNs were positive, or if regional recurrence occurred after a negative DSNB procedure without evidence of a new primary tumor or local recurrence. We calculated the false-negative rate according to the standard formula: false-negative rate = false-negative procedures/(true-positive procedures + false-negative procedures).

## Results

### Patient Characteristics and Histopathological Analysis

This study included 41 patients with penile cancer who underwent radioisotope-guided DSNB. The patient and tumor characteristics, including potential risk factors for postoperative complications after inguinal lymphadenectomy, such as obesity (BMI > 25), diabetes mellitus and cardiovascular disease (15, 44, 45), are listed in **Table 1**. Among the 76 groins that received DSNB, a total of 193 SLNs and 39 non-SLNs were removed. The median number of dissected LNs (SLNs + non-SLNs) per groin was 2.5 (interquartile range 2–4). Two patients had radioactive LNs located in the pelvis that were not dissected because they were not accessible through the incision of inguinal DSNB and considered second echelon LNs.

**Table 1:** Patient and tumor characteristics.

Characteristic	
Patients, *n*	41
Median age, years (interquartile range)	67 (61–73)
BMI, kg/m^2^, *n*	
< 25	13
> 25	28
Diabetes mellitus, *n*	
Yes	5
No	36
Cardiovascular disease, *n*	
Yes	26
No	15
Previous inguinal surgery, *n*	
Yes	5
No	36
Histological type of penile cancer, *n*	
Squamous cell carcinoma, usual type (with or without verrucous areas)	38
Papillary squamous cell carcinoma	1
Mixed squamous cell carcinoma	1
Papillary-basaloid carcinoma (HPV-related)	1
Tumor stage, *n*	
pT1G2	23
pT1G3	3
pT2G1	1
pT2G2	11
pT3G2	1
pT3G3	2
Surgical treatment of primary tumor, *n*	
Circumcision	4
Local excision at the penis shaft	2
Glansectomy with or without circumcision	16
Partial penectomy	19

Histopathological examination revealed that five of the 76 groins (6.6%) contained metastases. Three patients had unilateral and one patient had bilateral LN involvement. One patient with unilateral metastatic disease had two positive SLNs. Two of the four patients with LN metastases underwent primary DSNB and two underwent secondary DSNB. None of the non-SLNs harbored metastases. In accordance with the EAU guidelines, all groins with positive SLNs underwent secondary radical inguinal lymphadenectomy, which revealed three additional metastases in one patient with unilateral nodal involvement. This patient had a transient ischemic attack 1 month after radical inguinal lymphadenectomy and died a few months later, and no pelvic lymphadenectomy was therefore performed. None of the other patients showed further metastases at complementary radical inguinal lymphadenectomy. A summary of the histopathological findings is presented in **Table 2**. The histopathological results of the six groins that underwent radical or modified inguinal lymphadenectomy are given in **Table 3**.

**Table 2:** Histopathological results of DSNB.

Tumor stage	pN0 (*n* = 37)	pN+ inguinal (*n* = 4)	pN+ pelvic (*n* = 0)
pT1G2	21	2	0
pT1G3	3	0	0
pT2G1	1	0	0
pT2G2	10	1*	0
pT3G2	1	0	0
pT3G3	1	1	0

^*^Patient with bilateral LN metastases.

**Table 3:** Histopathological results of unilateral modified or radical inguinal lymphadenectomy in six patients.

Patient No.	Tumor stage	Type of lymphadenectomy	Groin	Reason	Number of dissected LNs	Number of positive LNs	LN status
2	pT3G3	Radical inguinal	Right	Suspicious LNs	3	0	pN0
25	pT1G3	Radical inguinal (+ pelvic)	Left	LN metastasis	5 (+ 11)	2 (+ 3)	pN+ inguinal (+ pelvic)
29	pT2G2	Modified inguinal	Left	Non-visualization	5	0	pN0
35	pT2G2	Radical inguinal (+ pelvic)	Left	Suspicious LNs	8 (+ 3)	2 (+ 0)	pN+ inguinal
38	pT1G2	Modified + radical inguinal	Left	Non-visualization	0 + 12	0 + 0	pN0
41	pT1G3	Radical inguinal	Right	LN metastasis	8	1	pN+ inguinal

### False-Negative Procedures and False-Negative Rate

The median follow-up was 70 (range 6–158, interquartile range 36–96) months. In total, three patients with bilateral negative DSNB developed regional recurrence. One patient underwent local tumor excision (R1 resection) and partial penectomy (R0 resection) before referral to our hospital for DSNB. Bilateral inguinal metastases and systemic metastatic disease were detected 32 months after DSNB and the patient died of penile cancer 4 months later. Another patient underwent partial penectomy simultaneously with DSNB. However, due to inaccessibility, a radioactive pelvic LN remained and the patient was diagnosed with left-sided inguinal and iliac LN metastases and pulmonary metastatic disease 7 months after DSNB. A third patient underwent radical circumcision and circular re-resection of the penile shaft skin in another hospital. He presented with an enlarged LN in the right groin 12 months after negative DSNB. This LN was dissected 2 months later and histopathological analysis revealed metastasis. The patient declined complementary radical inguinal lymphadenectomy because of the high morbidity risk, but he remained alive without evidence of disease at 13 months. The clinical and pathological characteristics of the false-negative patients are shown in **Table 4**. None of these patients had locally recurrent penile tumors or previous inguinal surgeries.

**Table 4:** Clinical and pathological characteristics of patients with false-negative DSNB results.

Patient No.	Age	Tumor stage	Surgical treatment of primary tumor	Timing of DSNB	Dissected LNs		LN status	Regional recurrence	Time to regional recurrence (months)	Distant recurrence	Status (time after recurrence, months)
					Left groin	Right groin					
17	81	pT2G2	Local excision + partial penectomy	Secondary	1 SLN + 1 non-SLN	1 SLN	pN0	Bilateral	32	Yes	Died of penile cancer (4)
39	66	pT3G2	Partial penectomy	Primary	1 SLN + 1 non-SLN	2 SLNs	pN0	Left groin	7	Yes	Unknown (0)
40	51	pT2G2	Radical circumcision + re-resection of penile shaft skin	Secondary	2 SLNs	1 SLN	pN0	Right groin	12	No	Alive without evidence of disease (13)

In summary, we encountered four true-positive and three false-negative patients (false-negative rate of 42.9%). However, two of the three false-negative patients had repetitive penile tumor resections prior to DSNB.

### Histopathological Re-Evaluation of SLNs in False-Negative Cases

Histopathological reexamination of the SLNs from the false-negative groins revealed normal lymphatic tissue. In the third false-negative patient (regional recurrence on the right side), one previously undetected micrometastasis (2 mm) was found in an SLN from the left groin. The results of the histopathological reexamination are summarized in **Table 5**.

**Table 5:** Results of histopathological reexamination of SLNs from false-negative patients.

Patient No.	False-negative groin	Metastasis	
		Left groin	Right groin
17	Left + right	No	No
39	Left	No	No
40	Right	Yes (micrometastasis, 2 mm)	No

### Follow-Up

During follow-up, four patients developed distant recurrences. Two of these patients had false-negative DSNB results (**Table 4**, patients 17 and 39). The third patient underwent unilateral DSNB (right groin, pN0) and unilateral radical inguinal and pelvic lymphadenectomy due to histologically confirmed lymph node metastasis (left groin) (**Table 3**, patient 25). 10 months after DSNB and radical inguinal lymphadenectomy, he presented with distant metastases. However, he did not show recurrent disease in inguinal or pelvic LNs of the right side and was therefore not classified false-negative. The fourth patient received unilateral DSNB (right groin, pN0) and unilateral modified inguinal lymphadenectomy due to non-visualization of SLNs (left groin, pN0) (**Table 3**, patient 29). 14 months later, he was diagnosed with retroperitoneal metastasis on the left side. The modified inguinal lymphadenectomy procedure was therefore considered false-negative.

Five other patients presented with local relapse and underwent further surgery (glansectomy or partial penectomy). One of these patients received a second bilateral DSNB, which did not reveal metastases. However, this patient was lost to follow-up after the hospital stay. Another patient with local recurrence was subsequently diagnosed with LN metastasis in the right groin and underwent radiotherapy.

Nine patients died during follow-up. The median follow-up of these patients was 33 (range 6-132, interquartile range 17–55) months. Two of the patients with distant metastases died of penile cancer (**Tables 3**, **4**, patients 17 and 25), one patient with systemic metastatic disease died 1 day after retroperitoneal tumor extirpation (R2 resection) due to pre-existing conditions (**Table 3**, patient 29), three patients died from causes unrelated to penile cancer (advanced lung cancer, renal cell carcinoma, pulmonary emphysema), and three other patients died of unknown causes.

### Complications

Postoperative complications after DSNB occurred in 12 groins, with a morbidity rate of 15.8% per inguinal region. Most complications were mild or moderate and non-invasive or invasive intervention was only required in six groins (7.9%). No patient died from complications. The DSNB-related complications graded according to Clavien–Dindo are shown in **Table 6**.

## Discussion

In this retrospective study, we investigated the reliability and morbidity of radioisotope-guided DSNB in a cohort of patients with penile cancer who underwent long-term follow-up in a tertiary referral hospital. This study represents the largest German series of penile cancer patients treated with DSNB to date. Notably, unlike other European countries, the treatment of penile cancer in Germany is not centralized. The current analysis revealed that DSNB was associated with a low complication rate of 15.8%. In total, we encountered four true-positive and three false-negative patients in our cohort of 41 patients; however, two of the three false-negative patients underwent repetitive penile tumor resections prior to DSNB.

The optimal management of regional LNs in cN0 patients with penile cancer has been controversial for many years. Clinical surveillance carries the risk of detecting metastases at a later stage, thereby compromising the oncological outcome (7, 8), whereas radical inguinal lymphadenectomy is associated with high morbidity and may result in overtreatment in 75%–80% of patients (14, 15). To reduce the morbidity associated with inguinal lymphadenectomy, the EAU guidelines recommend invasive LN staging by modified inguinal lymphadenectomy or DSNB in cN0 patients with ≥ T1G2 tumors (9).

In the present study, we reported a complication rate of 15.8% for radioisotope-guided DSNB, which was considerably lower than most of the published contemporary complication rates for radical inguinal lymphadenectomy ranging between 49% and 58% (14, 15, 46). Only one study by Koifman et al. revealed a lower complication rate of 10.3% (47). DSNB thus seems to be a suitable procedure for decreasing the morbidity risk in patients with cN0 penile cancer. Compared to the complication rate of 10–45% for modified inguinal lymphadenectomy, our complication rate for DSNB was similar (19, 21, 48). Previous studies showed high variability in complication rates for DSNB. A two-center study of 323 patients from the Netherlands and England found a morbidity rate of 4.7%, with most of the complications being transient and managed conservatively (49). Lam et al. found DSNB-related complications in 20 of 264 patients (7.6%), including lymphocele, wound infection, hematoma, penoscrotal lymphedema, and wound bleeding (35). In contrast, Dimopoulos et al. reported a higher overall morbidity rate of 21.4%, although, similar to the current study, most of the complications were categorized as Clavien-Dindo grade I–II (36). The apparently large variability in morbidity rates may be due to underreporting or differences in the definitions of complications (e.g., exclusion of complications without intervention). Although DSNB may avoid overtreatment in patients with penile cancer, it carries the risk of false-negative results, and a delayed detection of LN metastases may have a negative effect on patient survival (7, 8).

Several studies have investigated the reliability of DSNB in patients with penile cancer. The Netherlands Cancer Institute, which introduced DSNB in penile cancer, reported an initial false-negative rate of 19.2%–22% (30, 50). The initial DSNB procedure consisted of preoperative lymphoscintigraphy, sentinel node biopsy after peritumoral injection of blue dye, and histopathological examination. Detailed analysis of the false-negative cases led to several procedural modifications, including the addition of preoperative ultrasonography with fine needle aspiration cytology of suspicious LNs, followed by radical inguinal lymphadenectomy if the results were positive. In addition, scintigraphically non-visualized groins were surgically explored, the wound was intraoperatively palpated, and histopathological analysis was extended by serial sectioning and immunohistochemistry. These modifications reduced the false-negative rate to 4.8% per groin (30). A prospective study by Lam et al. analyzed 500 groins that underwent DSNB and found a false-negative rate of 5% per inguinal region (35). Two European multicenter studies reported false-negative rates of 7% and 10.8% per groin, respectively (37, 49). However, in line with our results, some other studies showed considerably higher false-negative rates. Using the isolated gamma probe technique, Gonzaga-Silva et al. found only one patient with LN metastases in a cohort of 27 patients, but three patients with a negative DSNB procedure developed regional recurrence during a mean follow-up of 36 months, resulting in a false-negative rate of 75% per patient. The authors concluded that the isolated gamma probe technique was not reliable for detecting LN metastases in patients with penile cancer (31). A study of 21 patients by Spiess et al. found a false-negative rate of 28.6% per groin (33). A recent review and meta-analysis of 27 articles on radioisotope-guided DSNB in penile cancer reported pooled sensitivity and negative-predictive values of 88% and 99%, respectively (51). The large variability in false-negative rates may be explained by the small patient cohorts, heterogeneity of DSNB protocols, and different levels of experience with the technique.

There are several possible reasons for false-negative DSNB results. One possibility is that histopathological analysis may fail to detect micrometastases; however, pathological reevaluation of the SLNs from the four false-negative groins in the current study revealed normal lymphatic tissue. False-negative results may also be due to tumor blockage, in which lymphatic drainage is obstructed by tumor cells leading to rerouting of the radioactive tracer to a “neo-SLN” (52). DSNB is thus not recommended in patients with palpable LNs because of the high risk of LN metastases and thus tumor blockage (9). False-negative procedures may also be caused by alteration of the lymphatic drainage as a result of the previous removal of the primary tumor. In the present study, two of the three patients with false-negative results had multiple primary tumor resections prior to DSNB. Graafland et al. investigated the reliability of postresection DSNB in a cohort of 40 patients and found no regional recurrence after a median follow-up of 28 months (38). In a study by Omorphos et al., one of 92 patients who underwent secondary DSNB developed regional recurrence during a median follow-up of 22 months, and the false-negative rate was 11.1% per patient (39). The results of these studies indicate that DSNB is reliable after previous removal of the primary tumor. In contrast, however, Fuchs et al. and Lützen et al. only observed regional recurrence after secondary but not after primary DSNB, which argues against this hypothesis (40, 41). Similarly, it is unclear whether DSNB is reliable in patients with local recurrence or previous inguinal surgeries who may have an altered lymphatic drainage, e.g. due to scarring. In the present study, none of the patients with locally recurrent tumors or previous groin surgeries developed regional recurrence. However, further studies with larger patient cohorts and long-term follow-up are needed to confirm or disprove the reliability of DSNB after surgical treatment of the primary tumor, local recurrence or previous inguinal surgeries.

In addition to the above reasons, several studies have suggested that the false-negative rate of DSNB depends on the protocol used. Dimopoulos et al. compared the results of 1- and 2-day protocols for DSNB in patients with penile cancer. The 1-day protocol resulted in harvesting of significantly more LNs than the 2-day protocol, with false-negative rates of 0% and 6.8%, respectively, suggesting that the 1-day protocol may be more reliable for the detection of LN metastases in patients with cN0 penile cancer (36). Moreover, preoperative ultrasonography and intraoperative palpation of the wound are suggested to improve the false-negative rate by identifying suspect LNs that are not visualized due to tumor blockage (30). In contrast, fine needle aspiration cytology is no longer recommended in cN0 patients because of its low sensitivity of 39% (9, 53). Many groups performed additional injection of blue dye to visualize the SLNs; however, several studies using a combination of ^99m^Tc nanocolloid and blue dye found no SLNs that were stained with blue dye but were not radioactive (32, 34, 54), suggesting that the addition of blue dye may not reduce the false-negative rate of DSNB. Our DSNB procedure included preoperative ultrasonography, ^99m^Tc nanocolloid injection on the day of surgery, and palpation of the exposed wound, and this protocol therefore cannot explain the high false-negative rate in our study.

Recent efforts have been made to further refine the DSNB technique in patients with penile cancer. The introduction of the hybrid radioactive and fluorescent tracer indocyanine green-^99m^Tc nanocolloid significantly improved the optical detection of SLNs compared with blue dye (55). Dell’Oglio et al. recently confirmed the reliability of indocyanine green-^99m^Tc nanocolloid for DSNB in a large cohort of 400 patients and reported false-negative rates of 10% per patient and 8.9% per groin (56). Moreover, initial results indicated that magnetometer-guided DSNB using superparamagnetic iron oxide nanoparticles was a feasible, radiation-free technique for the identification of SLNs in penile cancer (57, 58). Another recent study investigated the use of intraoperative freehand magnetic particle imaging together with a hybrid indocyanine green–superparamagnetic iron oxide nanoparticle tracer for intraoperative SLN detection (59). The feasibility of this method was confirmed in *ex vivo* human skin transplants and in a porcine model, but the results need to be verified in human patients. Further refinements of the DSNB technique will hopefully reduce the false-negative rate of the procedure in the future. Apart from that, Choo et al. recently reported that adding postoperative adjuvant concurrent radiotherapy and chemotherapy may have a therapeutic benefit and may help to further improve survival in patients with penile cancer and regional LN metastases (60).

The present study had some limitations. One limitation was the retrospective nature of the study with all its drawbacks, such as a possible information bias due to incomplete data in medical records. Moreover, our analysis relied on a single center and included a relatively small number of patients because of the low incidence of penile cancer and the non-centralized treatment of penile cancer in Germany. These issues should be taken into account when interpreting the results of the present study. Only nine groins that underwent DSNB contained LN metastases (true-positive + false-negative procedures), and a single false-negative event thus had a great impact on the false-negative rate. Nonetheless, our study represents the largest German series of the use of DSNB in patients with penile cancer published to date. The study was also limited by the follow-up time; although 86.1% of regional recurrences occur within 2 years after primary treatment (61), we cannot rule out the possibility that further false-negative procedures would come to light in the future.

In conclusion, the results of this study suggest that radioisotope-guided DSNB may reduce the morbidity of inguinal lymphadenectomy in patients with cN0 penile cancer. However, DSNB and primary tumor resection should be performed simultaneously to avoid false-negative results. Recent advances, such as new tracers and imaging techniques, may help to further reduce the false-negative rate of DSNB.

**Table 6:** DSNB-related complications.

Complication	No. of DSNB procedures (*n* = 76)	Clavien–Dindo classification, grade
Lymphocele/seroma (no intervention)	4	I
Hematoma (no intervention)	2	I
Wound infection requiring antibiotics	3	II
Lymphocele requiring drainage	1	IIIa
Wound infection requiring revision operation	2	IIIb
Total (%)	12 (15.8%)	

## Data Availability Statement

The raw data supporting the conclusions of this article will be made available by the authors, without undue reservation.

## Ethics Statement

The studies involving human participants were reviewed and approved by Medical Ethics Committee of the University of Oldenburg. The patients/participants provided their written informed consent to participate in this study.

## Author Contributions

AW and FW conceived the study. LN, AV, L-MM, and AW acquired and analyzed the data. All authors contributed to the interpretation of data. R-PH carried out the histopathological reexaminations of the lymph nodes. LN wrote a first draft of the manuscript. AW, FW, AV, BM, and SE revised the manuscript. All authors contributed to the article and approved the submitted version.

## Conflict of Interest

The authors declare that the research was conducted in the absence of any commercial or financial relationships that could be construed as a potential conflict of interest.

## Publisher’s Note

All claims expressed in this article are solely those of the authors and do not necessarily represent those of their affiliated organizations, or those of the publisher, the editors and the reviewers. Any product that may be evaluated in this article, or claim that may be made by its manufacturer, is not guaranteed or endorsed by the publisher.
